# Enhanced expression of recombinant beta toxin of *Clostridium perfringens* type B using a commercially available *Escherichia coli* strain

**DOI:** 10.4102/ojvr.v83i1.1136

**Published:** 2016-06-30

**Authors:** Fatemeh Bakhshi, Reza Pilehchian Langroudi, Bahram Golestani Eimani

**Affiliations:** 1 Department of Biology, Islamic Azad University, Urmia branch, Urmia, Iran; 2 Razi Vaccine and Serum Research Institute, Agricultural Research, Education and Extension Organization (AREEO), Alborz, Karaj, Iran

## Abstract

*Clostridium perfringens* beta toxin is only produced by types B and C and plays an important role in many human and animal diseases, causing fatal conditions that originate in the intestines. We compared the expression of *C. perfringens* type B vaccine strain recombinant beta toxin gene in the *Escherichia coli* strains Rosetta^TM^(DE3) and BL21(DE3). The beta toxin gene was extracted from pJETβ and ligated with pET22b(+). pET22β was transformed into *E. coli* strains BL21(DE3) and Rosetta^TM^(DE3). Recombinant protein was expressed as a soluble protein after isopropyl β-D-1-thiogalactopyranoside (IPTG) induction in strain Rosetta^TM^(DE3) but not in BL21(DE3). Expression was optimised by growing recombinant cells at 37 °C and at an induction of 0.5 mM, 1 mM, 1.5 mM IPTG. Expression was evaluated using sodium dodecyl sulfate Polyacrylamide gel electrophoresis (SDS-PAGE). The recombinant protein was purified via Ni-NTA and was analysed using western blot. We concluded that *E. coli* strain Rosetta^TM^(DE3) can enhance the expression of *C. perfringens* recombinant beta toxin.

## Introduction

*Clostridium perfringens,* an anaerobic Gram-positive, rod-shaped bacterium, is able to form environmentally resistant spores. *Clostridium perfringens* produces seventeen toxins. Four of them – iota, alpha, beta and epsilon – are major toxins and are used for classifying *C. perfringens* into five types – A, B, C, D and E (Nilo 1980). Beta toxin is only produced by types B and C and plays an important role in many human and animal diseases via pore formation in the endothelial cell membrane (Michlard *et al*. 2009; Nagahama *et al*. 2015). *Clostridium perfringens* beta toxin (CPB) forms a multimeric transmembrane pore in the endothelial cell membrane and is the cause of cell lysis (Steinthorsdottir *et al*. 2000). *Clostridium perfringens* has a circular chromosome of approximately 3.6 mega-base pairs with a low content of G+C (about 25%) (Canard *et al*. 1992; Casjens 1998). The genome *C. perfringens* includes extra chromosomal genetic elements in the form of plasmid and phage-encoded mobile genes that can vary in size and composition (Bruggemann 2005; Cavalcanti *et al*. 2004). *Clostridium perfringens* beta toxin gene (*cpb*), which encodes a protein made up of 309 amino acids, is located on the different large plasmids that are carried by types B and C (Rokos, Rood & Duncan 1978). Expression of CPB in *Escherichia coli* has been shown and a molecular analysis revealed that it has sequence homology with alpha-toxin, gamma-toxin and leukocidin of *Staphylococcus aureus* (Hunter *et al*. 1993).

In the present study, the beta toxin gene of the *C. perfringens* vaccine strain (CWB CN228) was expressed in *E. coli* and purified proteins were analysed. This vaccine strain was isolated in Iran (Brooks & Entessar 1957), and its beta toxin gene was sequenced, described and deposited in GenBank HQ179547.1.

## Materials and methods

The expressions of *C. perfringens* type B vaccine strain recombinant beta toxin gene in the *E. coli* strains Rosetta^TM^(DE3) and BL21(DE3) were compared. The methods used have been described elsewhere (Pilehchian Langroudi *et al*. 2011).

Plasmid pJET1.2/blunt, Pfu DNA polymerase, dNTPs, T4 DNA ligase, NdeI and XhoI REs and GeneRuler™, 100 bp Plus DNA size markers, PageRuler™, and extraction kit, SDS, Proteinase K, lysozyme and plasmid extraction kit were purchased from Fermentas (Thermo Scientific™ Germany). High Pure PCR Product Purification Kit for DNA fragments recovery was purchased from Vivantis Technologies Sdn Bhd (Selangor, Malaysia). Sheep primary antibody and conjugate anti-sheep Horseradish_peroxidase (HRP) were purchased from DAKO Company (Glostrup, Denmark). *Clostridium perfringens* type B vaccine strain (CWB CN228), *E. coli* strain TOP10, which was applied as a cloning host, and *E. coli* strains BL21(DE3) and Rosetta™(DE3) (Novagen, Merck Millipore, Germany) as expression hosts were prepared at the Razi Institute. *Clostridium perfringens* was cultured anaerobically in the liver extraction media at 37 °C for 5 h after the centrifugation supernatant was removed and discarded; the whole genomic DNA was extracted using the phenol–chloroform method. The gene *cpb* was amplified via PCR using one pair of specific primers consisting of *Nde*I at the 5, end of the forward and *Xho*I at the 3, end of the reverse primers. (Pilehchian Langroudi *et al*., 2011). *Pfu* DNA polymerase was used for amplification of the blunt-end PCR product. After ligation of the *cpb* gene into the pJET1.2/blunt and producing pJETβ (pJET1.2/blunt beta) recombinant vector, the *E. coli* strain Top10 competent cell was transformed using pJETβ, and the screening of recombinant clones was done via antibiotic resistance and colony PCR. Nucleotide sequencing was carried out via Source Bioscience Co. (Berlin, Germany). The pJETβ recombinant vector was purified from *E. coli*/TOP10/pJETβ cells and was digested using *Nde*I and *Xho*I. After digestion, the *cpb* gene was extracted from the gel and was purified. pET22b(+) was digested using the same enzymes, was purified from the electrophoresis gel and was ligated using *cpb* gene. The ligation product was applied on the 1% electrophoresis agarose gel to show the formation of recombinant plasmid. *Escherichia coli* strains BL21(DE3) and Rosetta™(DE3) were selected as expression hosts. After preparation of the competent cells, they were transformed using pET22β [pET22b(+) beta] expression vector so that *E. coli*/BL21/pET22β and *E. coli*/Rosetta™/pET22β were produced. To confirm the presence of the recombinant pET22β expression vector, recombinant cell screening was done, as previously described (Pilehchian Langroudi *et al*., 2011). Colony PCR was done using recombinant individual *E. coli* colonies. After purification, the recombinant pET22β expression vector was sequenced.

### Expression of *Clostridium perfringens* beta toxin protein

Recombinant cells were cultured in LB/Amp media and were incubated at 37 °C to OD_600_ = 0.6–0.7. To induce protein expression, 0.5 mM Isopropyl β-D-1-thiogalactopyranoside (IPTG) was added and growth was continued for 18 h; in additional trials, different concentrations of IPTG (0.5 mM, 1 mM and 1.5 mM) were used. Effects of a temperature gradient (25 °C, 31 °C and 37 °C) and time variation (3, 6 and 18 h) were considered. The expressed protein analysis was performed using sodium dodecyl sulfate Polyacrylamide gel electrophoresis (SDS-PAGE). Negative controls including of the *E. coli*/BL21/pET22 and *E. coli*/Rosetta^TM^/pET22 were used for each analysis. The recombinant beta protein, which contains a 6-His tag at the C-terminal, was purified with Ni-NTA resin. The bacterial pellet was suspended in the lysis buffer and the cells were lysed with sonication repeated six times on ice for a duration of 5 min. The cell lysate was centrifuged at 13 680 g, and the clarified supernatant was loaded on Ni-NTA resin at the flow rate of 1 mL/min. The column was washed with five volumes of wash buffer, and finally, the protein was eluted by adding elution buffer, as previously described (Pilehchian Langroudi *et al*. 2013). The purified protein was analysed using SDS-PAGE and western blot. The protein concentration was determined using a standard procedure, as previously described (Bradford 1976).

## Results

**FIGURE 1:**
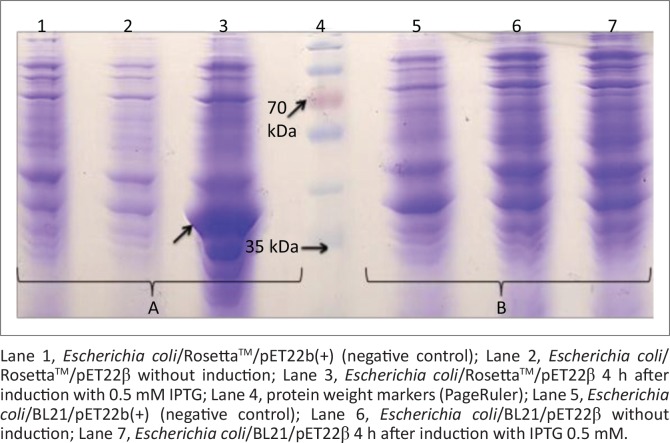
Comparison of beta toxin expression in (A) *Escherichia co-li*/Rosetta^TM^/pET22β and (B) *Escherichia co-li*/BL21/pET22β.

**FIGURE 2:**
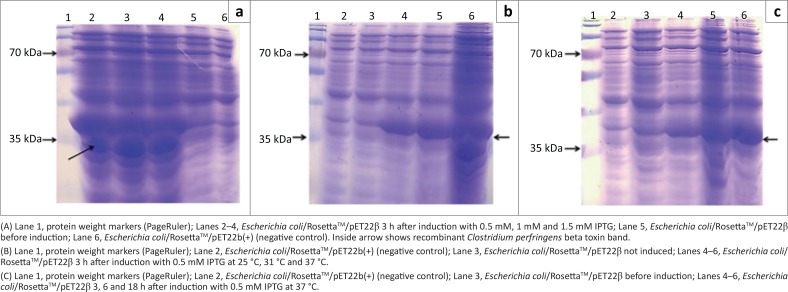
(a) Effects of IPTG concentration on expression. (b) Effects of different temperature on expression. (c) Effects of time on recombinant *Clostridium perfringens* beta toxin expression.

**FIGURE 3:**
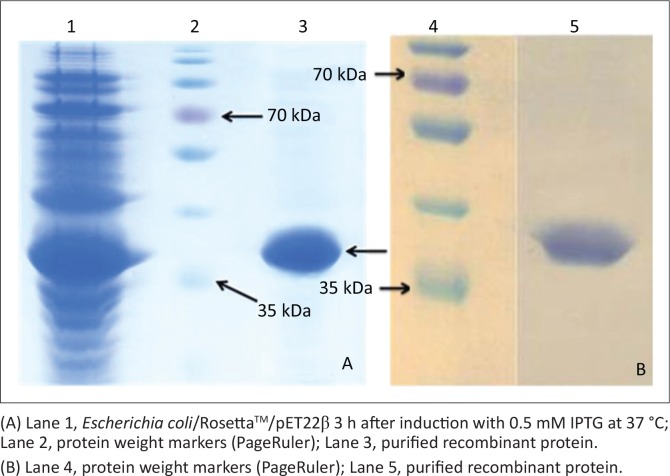
Sodium dodecyl sulfate polyacrylamide gel electrophoresis and western blot analysis of purified recombinant beta toxin. (A) Sodium dodecyl sulfate polyacrylamide gel electrophoresis. (B) Western blot using sheep primary antibody and conjugate anti-sheep Horseradish_peroxidase.

The electrophoresis result showed that genomic DNA was extracted and the PCR analysis revealed that the *cpb* gene was amplified. After successful production of the recombinant pJETβ cloning vector and the *E. coli*/TOP10/pJETβ cells and also successful subcloning of the pET22β, *E. coli* strains BL21(DE3) and Rosetta^TM^(DE3) cells were transformed using this expression vector. The colony PCR result showed the presence of the recombinant pET22β plasmids in both recombinant *E. coli* colonies. Sequencing revealed that the inserted gene was consistent with the *cbp* gene in GenBank (HQ179547.1). After expression, the SDS-PAGE analysis showed that the recombinant protein was well expressed between 2 and 4 h after induction via 0.5 mM IPTG in recombinant *E. coli/*Rosetta^TM^(DE3) but not in *E. coli/*BL21 (Figure 1). The use of different concentrations of IPTG had no significant effect on protein expression, but the effects of temperature and time on expression levels were significant, and in the case of time the effect continued up to 18 h (Figure 2). Recombinant protein was purified using Ni-NTA resin, and the result showed a very sharp band of the purified protein as an approximately 35-KDa using SDS-PAGE and western blot analysis (Figure 3).

## Discussion

Iranian variant type B was isolated in 1954 from intestinal contents of sheep and goats. The three strains of *Clostridium welchii* type B isolated were different from the classical type B strains in their production of kappa and non-production of lambda and hyaluronidase toxins. Two of the strains were isolated from young goats and the other from an adult sheep (Brooks & Entessar 1957). By now, some vaccines have been manufactured using the secreted CPB protein of *C. perfringens* types B and C. It has been about five decades since the vaccine against *C. perfringens* types B and C, CPB protein was produced in Iran (Pilehchian langroudi 2015) Genetic engineering, helping to produce recombinant protein that as a good alternative to native toxins belonging to the *C. perfringens* species (Nijland *et al*. 2007). In 1998, CPB of *C. perfringens* was expressed and secreted in *Bacillus subtilis* in the form of mutant protein (beta toxoid). The mutation of two amino acids that were located in the membrane binding region affected the lethal dose in mice (Steinthorsdottir *et al*. 1998). Previously, a genetic construct containing *C. perfringens* epsilon toxin protein (ETX) and CPB genes was produced in Iran. The fusion gene was expressed as a soluble protein in the *E. coli* strain Rosetta™ and its immunogenicity was studied in mice. (Pilehchian Langroudi *et al*. 2013).

In the present study, recombinant plasmid pET22bβ was transformed into *E. coli* strains BL21(DE3) and Rosetta^TM^(DE3). The SDS-PSAGE analysis showed no CPB band from *E. coli*/BL21/ pET22β but showed a CPB band from *E. coli*/Rosetta^TM^/pET22β. The sequencing analysis of purified pET22β revealed that the inserted gene had 987 bp, demonstrating a 99% – 100% identity with cpb genes that were previously deposited in GenBank. The recombinant toxin was expressed 30 min after induction with IPTG and continued for 18 h. It has been reported that the expression of recombinant beta toxin in *E. coli* started 30 min after induction with 0.5 mM IPTG and continued up to 6 h (Steinthorsdottir *et al*. 1995). It was revealed that different concentrations of IPTG had no obvious effect on the protein expression level. A previous report showed that there was no more expression after induction beyond 1 mM IPTG (Goswami *et al*. 1996). The optimal thermal condition for protein expression is 37 °C. At present, accessing recombinant bacterium and manufacturing a recombinant vaccine is possible. (Michlard *et al*. 2009) The strain used in this work was *C. perfringens* type B vaccine strain, which is the Iran variant of this species (Brooks & Entessar 1957). Although the beta toxin gene is significantly similar to other genes, it is unique for Iran. This strain is one of the active ingredients in the tetravalent enterotoxaemia vaccine in Iran, so it is very important that we use its beta toxin protein in the future recombinant vaccine.

## Conclusion

We concluded that pET22b(+) and *E. coli* strain Rosetta^TM^(DE3) are suitable expression vectors and hosts that can enhance the expression and production of *C. perfringens* recombinant beta toxin. Therefore, the *E. coli/*Rosetta^TM^/pET22β clone could be used for further research on recombinant vaccine production.
